# Endothelial cell markers reflecting endothelial cell dysfunction in patients with mixed connective tissue disease

**DOI:** 10.1186/ar2999

**Published:** 2010-05-06

**Authors:** Pal Soltesz, Daniel Bereczki, Peter Szodoray, Maria T Magyar, Henrietta Der, Istvan Csipo, Agota Hajas, Gyorgy Paragh, Gyula Szegedi, Edit Bodolay

**Affiliations:** 1 3rd Department of Medicine, Medical and Health Science Center, University of Debrecen, Moricz Zs. Str. 22, Debrecen 4032, Hungary; 2 Department of Neurology, Semmelweis University of Budapest, Balassa Str. 6, Budapest 1083, Hungary; 3 Institute of Immunology, Rikshospitalet, University of Oslo, Sognsvannsveien Str. 20, Oslo 0027, Norway; 4 Department of Neurology, Medical and Health Science Center, University of Debrecen, Moricz Zs. Str. 22, Debrecen 4032, Hungary; 5 1st Department of Medicine, Medical and Health Science Center, University of Debrecen, Nagyerdei Str. 98, Debrecen 4032, Hungary

## Abstract

**Introduction:**

The aim of the present study was to investigate the association between cardiovascular risk factors and endothelial dysfunction in patients with mixed connective tissue disease (MCTD) and to determine which biomarkers are associated with atherosclerotic complications, such as cardiovascular disease.

**Methods:**

Fifty MCTD patients and 38 healthy age-matched and sex-matched controls were enrolled in this study. In order to describe endothelial dysfunction, we assessed flow-mediated dilation (FMD), nitrate-mediated dilation (NMD) and carotid artery intima-media thickness (IMT). We investigated FMD of the brachial artery after reactive hyperemia and NMD after sublingual nitroglycerin administration, while the IMT of the common carotid artery was determined by ultrasound. Anti-U_1 _ribonucleoprotein (anti-U_1_RNP) antibodies, anti-cardiolipin (anti-CL) antibodies, anti-endothelial cell antibody (AECA) and endothelial cell markers, such as soluble thrombomodulin (TM) and von Willebrand factor antigen (vWFAg), were assessed.

**Results:**

The endothelium-dependent vasodilation (FMD) was significantly impaired in patients with MCTD, as compared with controls (%FMD: 4.7 ± 4.2% vs. 8.7 ± 5.0%; *P *< 0.001), while the percentage NMD did not differ (%NMD: 14.3 ± 6.6% vs. 17.1 ± 6.7%; *P *= 0.073). Mean carotid IMT values were higher in patients than in controls (IMT: MCTD, 0.64 ± 0.13 mm vs. controls, 0.53 ± 0.14 mm; *P *< 0.001). FMD negatively correlated with disease duration, the levels of apolipoprotein A_1_, the paraoxonase-1 activity, and systolic blood pressure in MCTD patients. The percentage FMD was significantly lower in MCTD patients with cardiovascular diseases (CVD), than in those without CVD (%FMD: 3.5 ± 2.9 vs. 5.8 ± 4.8, *P *< 0.0002), while percentage NMD did not differ between patients with and without CVDs. Serum levels of autoantibodies (anti-U_1_RNP, AECA and anti-CL) were significantly higher in MCTD patients and differed between MCTD patients with and without CVD. Endothelial cell markers such as soluble TM (12.2 ± 8.1 ng/ml vs. 3.2 ± 1.3 ng/ml; *P *< 0.001) and vWFAg (224.1 ± 115% vs. 89.4 ± 27.1%, *P *< 0.001) were the highest in MCTD patients with CVD.

**Conclusions:**

FMD is a reliable sensitive marker of endothelial cell dysfunction in MCTD. Beside the traditional risk factors, anti-U_1_RNP, AECA and anti-CL antibodies may be important not only in the pathogenesis of MCTD but in the induction of endothelial cell activation, and may play crucial roles in the development of early atherosclerosis in MCTD.

## Introduction

Systemic autoimmune diseases, such as systemic lupus erythematosus (SLE), rheumatoid arthritis or systemic sclerosis, are chronic inflammatory disorders - signified by complex interactions amongst traditional and nontraditional disease-related phenomena, including inflammation, dyslipidemia, thrombotic events, and humoral autoimmune processes [1-3]. Mixed connective tissue disease (MCTD) is also a chronic inflammatory systemic autoimmune disease, characterized by high titers of anti-U_1 _ribonucleoprotein (anti-U_1 _RNP) antibodies [4-7].

The frank tissue inflammation and proliferating vascular arteriopathy is a specific feature of MCTD. Proliferative vasculopathy involves the small and large arteries in various organs. The lung is the most frequent predilection place of the vascular damage, however, and may eventually lead to pulmonary arterial hypertension (PAH) [8]. Our group and others found that PAH might be associated with coexistent antiphospholipid and anti-endothelial cell antibodies (AECAs) [9,10]. In our previous study we found that AECA provokes the surface expression of E-selectin and the activation of endothelial cells [11]. In sera of patients with PAH, high concentrations of thrombomodulin (TM) and von Willebrand factor antigen (vWFAg) secreted from Weibel-Palade bodies imply an activated state of the endothelial cells. TM - an endothelial high-affinity receptor for thrombin - has an anticoagulant effect, activating the protein C system [12]. Soluble TM can be measured in peripheral blood, and an elevated level of soluble TM is a marker of endothelial injury.

Previously we described high levels of total serum cholesterol and reduced paraoxonase-1 (PON1) concentrations and activity in patients'sera [13]. PON1 has an antioxidant function, and it is a key factor in atherosclerotic events [13].

These data suggest that patients with MCTD have the traditional risk factors for the early development of atherosclerosis. The connection between endothelial cell damage and atherosclerosis in MCTD, however, has not been described previously.

Endothelial dysfunction is both an early marker of vascular diseases and a facilitating factor in the development of atherosclerosis [14-16]. Flow-mediated dilatation (FMD) of the brachial artery is a reliable and reproducible non-invasive tool to evaluate endothelial function [17-19]. The administration of sublingual nitrates is a good test to examine the vasodilatatory effect of an exogenous source of nitric oxide. The increase in carotid intima-media thickness (IMT) is a useful marker of systemic subclinical atherosclerosis and a strong predictor of subsequent myocardial infarction and stroke [20-22].

Since endothelial dysfunction represents an early stage of atherogenesis, the aim of this study was to determine whether impaired FMD amongst other biomarkers of endothelial dysfunction, is characteristic of patients with MCTD.

We aimed to determine which factors are associated with cardiovascular events in MCTD. We also investigated the endothelial cell functions, especially FMD, together with the circulating endothelial cell markers, soluble TM and vWFAg, reflecting the state of activation/damage of the endothelium. Finally, we investigated the relationship between peripheral endothelial dysfunction and carotid IMT.

## Materials and methods

### Patients

Fifty women with MCTD, treated and followed-up at the 3rd Department of Internal Medicine, University of Debrecen, were enrolled in the present study. Of these, 23 women (46%) had a history of cardiovascular diseases (CVDs). CVD was diagnosed if: the patient had acute myocardial infarction, or had ECG signs of myocardial infarction, recorded semi-annually; the coronary disease was treated with coronary bypass operation or angioplasty; or angina was verified by angiography and/or the ischemic alterations were verified by a non-invasive test or tissue Doppler's examination. Twenty-seven female MCTD patients without CVD were included and denoted as the MCTD/CVD-negative group.

All patients fulfilled the criteria for MCTD according to Alarcon-Segovia and Villarreal [23]. Clinical disease activity was assessed by the systemic lupus activity measure (SLAM) retrospectively from the patients' reports [24,25]. A SLAM value >6 was considered high disease activity.

MCTD patients were followed-up every 4 months at the outpatient clinic. Diagnostic procedures for MCTD included X-ray scan, lung function tests, electromyography and electroneurography. Esophageal involvement was detected by radionuclid esophageal transit scintigraphy and radiographic passage using gastrographin, or barium. Myositis was confirmed by muscle biopsy and/or electromyogram and creatinine kinase elevation. Raynaud's phenomenon was assessed by a positive color chart or cold test, and morphological abnormalities were assessed by nailfold capillary microscopy (Nikon Corp., Vienna, Austria).

Abnormal findings were assessed, as earlier described by Maricq [26]. Abnormal findings were as follows: number of loops in a linear 1 mm width, enlargement of capillary loops, or presence of bushy capillaries and avascular areas. Avascularization was assessed by methods described by Lee and colleagues [27]. The scleroderma pattern was characterized by nailfold microhemorrhages, enlarged loops, and avascular areas.

In laboratory analyses, we assessed the erythrocyte sedimentation rate (ESR), high-sensitivity C-reactive protein (hs-CRP), the routine blood count, and the renal and liver function; urine analysis was also performed. Traditional risk factors for cardiovascular disease such as age, body mass index (BMI), fasting plasma glucose, plasma lipid levels, as well as blood pressure were recorded.

Thirty-eight age-matched and BMI-matched female control subjects were also enrolled in the study. Exclusion criteria included known CVD, diabetes mellitus, obesity (BMI >30), and infection. Patients and controls had not consumed alcohol or taken vasoactive drugs within the past 24 hours. No patients received lipid-lowering therapies 48 hours before the study.

Cumulative lifetime corticosteroid dose (prednisone equivalent/grams) was calculated from hospital records. Twenty-two out of 50 patients with MCTD received nonsteroidal anti-inflammatory drugs, 10 patients had the combination of corticosteroids (CS) and salazopyrine, and 11 patients received a combination therapy of CS and methotrexate, while seven patients received CS therapy alone.

The protocol was in full compliance with the Good Clinical Practices, the Declaration of Helsinki, and the guidelines of the Medical and Health Science Centre of the University of Debrecen. The protocol has been approved by the institutional ethics committee (Regional and Institutional Ethics Committee, Medical and Health Science Centre, University of Debrecen). Written informed consent was obtained from all patients and healthy controls.

### Immunoserological investigations

The detection of antinuclear antibodies on the HEp2 cell line was carried out by indirect immunofluorescence. The serum concentrations of autoantibodies were analyzed by ELISA according to the manufacturer's instructions: anti-Sjögren syndrome-associated antigen A antibodies, anti-Sjögren syndrome-associated antigen B antibodies, anti-Sm antibodies, anti-Sc170 antibodies, anti-dsDNA antibodies, anti-cardiolipin (anti-CL) antibodies (Cogent Diagnostic, Edinburgh, UK), and anti-U_1_RNP antibodies (Pharmacia and Upjohn, Freiburg, Germany). Absorbances were measured by a Labsystems Multiscan MS ELISA reader at 492 nm (Labsystems Oy, Helsinki, Finland). The concentrations of samples were determined using a standard curve obtained from the optical densities of standards with known concentration.

### Endothelial cell markers

Thrombomodulin levels were measured by ELISA using commercial reagents according to the manufacturer's instructions (Diagnostic Stago, Asnieres, France). All assays were performed in duplicate. The intra-assay and interassay coefficients of variation for all ELISA assays were <5% and <10%, respectively.

The assessment of AECAs was performed on endothelial cells from human umbilical cord veins, employing an ELISA method, described previously in detail [11].

### Laboratory examinations

The total cholesterol concentration was determined spectrophotometrically. Triglyceride and high-density lipoprotein cholesterol (HDL-C) was measured with the immune turbidimetric method. Low-density lipoprotein cholesterol (LDL-C) was measured by homogenous, enzymatic, colorimetric assay (Roche LDL-C plus 2nd generation; Roche, Basel, Switzerland).

Apolipoprotein A_1 _(ApoA_1_) and apolipoprotein B were assessed with the Orion Diagnostica kit (Orion Diagnostica, Espoo, Finland), which employs an immune-nephelometric method. PON1 activity was measured spectrophotometrically [13].

### Endothelium-dependent (flow-mediated) and endothelium-independent (nitrate-mediated) vasodilation

Endothelium-dependent vasodilation was assessed with a 7.5-MHz linear array transducer (Hewlett-Packard Sonos 5500; Soma Technology Inc., Bloomfield, CT, USA) by scanning the brachial artery in longitudinal sections, as published previously [28]. Endothelial function testing was performed by the same person (HD) and the evaluation was carried out offline by a digital software technique (AVITA; Gtech Information Systems, Naperville, IL, USA), as described previously in detail [29].

The inter-observer analysis found the variability on 20 patients to be 8.95%. The intra-observer analysis was performed on 10 healthy individuals three times, with a 30-minute interval between the analyses. The intra-observer variability was 4.6%. We performed the variation coefficient for baseline diameter in 20 cases, and it was 0.86%; the accuracy of the method is therefore appropriate according to the international recommendation [30].

### Carotid duplex ultrasound investigations; measurement of carotid artery intima-media thickness

Ultrasound examinations were performed immediately after bloodsampling with the color-coded Hewlett Packard SONOS 5500 (Soma Technology Inc.) carotid duplex equipment with a 7.5 MHz linear transducer. The investigation included longitudinal and transverse examinationsof the carotid arteries. Measurements of IMT were performed at about 10 mm proximalto the carotid bulb, or at 20 mm proximal to the flow divider. The IMTwas measured as the distance between the leading edge of the first echogenicline (lumen-intima interface) and the second echogenic line(upper layer of the adventitia) in the far (deeper) artery wall. All measurements were performed at the end of the heart cycle, when the transducerwas in the mediolateral direction [31]. Offline analysis was performed by digital video images (AVITA; Gtech Information Systems). Measurements were performedin both carotid arteries and the larger of the two values was used for analysis.

### Statistical analysis

Normality of continuous variables was evaluated by the Shapiro-Wilk test. As most variables were not normally distributed, the Mann-Whitney test was used to compare controls and MCTD patients. Factors that differed significantly between patients and controls in univariate tests were entered in a general linear model to test whether these factors are independent predictors of FMD, nitrate-mediated dilation (NMD) and IMT. Statistica for Windows (StatSoft, Tulsa, OK, USA) was used for data analysis. Correlations were determined using Spearman correlation coefficient. Statistical significance was assumed when *P *< 0.05.

## Results

In the patient group the mean ± standard deviation age at the time of investigation was 50.2 ± 10.0 years (range: 17 to 69 years) and the disease duration was 9.56 ± 6.8 years (range: 3 to 26 years). Patients with MCTD and the control group were similar with regards to age, systolic and diastolic blood pressures, triglyceride, LDL-C, HDL-C, as well as BMI (Table 1). The total cholesterol (*P *< 0.047), the PON1 activity (*P *< 0.001) and ApoA_1 _levels (*P *< 0.001) were significantly lower in MCTD patients than in the controls, while there was no difference in apolipoprotein B concentrations between patients with MCTD and controls (*P *= 0.693). The ApoA_1 _and PON1 activities were lower in the MCTD/CVD^+ ^and MCTD/CVD^- ^patient groups compared with controls. The ESR and hs-CRP levels were significantly higher in patients with MCTD and in the MCTD/CVD^+ ^and MCTD/CVD^- ^groups compared with healthy controls.

**Table 1:** Baseline characteristics of patients

Feature	MCTD^a^	Controls	*P *value, MCTD vs. controls	MCTD^b ^CVD^+^	MCTD CVD^-^	*P *value, MCTD/CVD^+ ^vs. MCTD/CVD^-^
*n*	50	38		23	27	
Age at investigation (years)	50.2 ± 10.0	50.4 ± 10.3	0.936	50.1 ± 10.7	50.2 ± 9.5	0.99
Disease duration (years)	9.56 ± 6.8 (3 to 26)	-	-	10.3 ± 6.6 (3 to 26)	8.9 ± 6.9 (3 to 25)	0.481
SBP (mmHg)	137.4 ± 21.1	132.9 ± 19.6	0.367	144.7 ± 19.5	131.1 ± 20.6	0.037
DBP (mmHg)	90.9 ± 15.9	86.6 ± 15.3	0.215	88.5 ± 14.9	92.9 ± 16.8	0.299
BMI (kg/m^2^)	24.2 ± 1.3	23.2 ± 1.1	0.77	23.9 ± 1.1	24.81.9	0.383
Smoking						
Former smokers	5 (10%)	5 (13.1)	0.7401	2 (8.6)	3 (11.1)	1.0
Nonsmokers	45 (90%)	33 (86.8%)	0.7401	21 (91.3)	24 (88.8)	1.0
Serum triglyceride (mmol/l)	1.611 ± 0.76	1.6 ± 0.79	0.755	1.5 ± 0.82	1.72 ± 0.70	0.3367
Total cholesterol (mmol/l)	5.94 ± 1.18	5.47 ± 1.03	0.047	5.8 ± 1.2	6.06 ± 1.13	0.108
HDL-C (mmol/l)	1.69 ± 0.5	1.68 ± 0.49	0.943	1.56 ± 0.48	1.8 ± 0.5	0.22
LDL-C (mmol/l)	3.62 ± 1.15	3.23 ± 0.88	0.148	3.52 ± 1.19	3.7 ± 1.12	0.333
ApoA_1 _(g/l)	1.31 ± 0.36	1.72 ± 0.47	<0.001	1.27 ± 0.3	1.33 ± 0.4	0.003
ApoB (g/l)	0.95 ± 0.31	0.91 ± 0.13	0.693	0.98 ± 0.38	0.89 ± 0.15	0.92
Paraoxonase-1 activity	113.6 ± 70.6	187.4 ± 68.3	<0.001	84.32 ± 69.66	138.7 ± 62.3	<0.001
hs-CRP (mg/l)	15.2 ± 9.62	1.44 ± 0.99	<0.001	22.1 ± 9.78	9.36 ± 3.96	<0.001
ESR (mm/hour)	20.2 ± 17.8	8.3 ± 3.9	<0.001	26.5 ± 17.6	14.4 ± 16.2	0.014

ApoA_1_, apolipoprotein A_1_; ApoB, apolipoprotein B; BMI, body mass index; CVD, cardiovascular disease; DBP, diastolic blood pressure; ESR: erythrocyte sedimentation rate; HDL-C, high-density lipoprotein-cholesterol; hs-CRP: high-sensitivity C-reactive protein; LDL-C, low-density lipoprotein-cholesterol; MCTD, mixed connective tissue disease; SBP, systolic blood pressure. ^a^Mann-Whitney U test. ^b^Kruskal-Wallis analysis of variance.

The involvement of various organs is summarized in Table 2. The presence of Raynaud's phenomenon (*P *< 0.006), PAH (*P *= 0.0141), and secondary antiphospholipid syndrome (*P *= 0.015) were significantly more frequent in MCTD/CVD^+ ^patients than in the MCTD/CVD^- ^patient group.

**Table 2:** Clinical symptoms of mixed connective tissue disease patients with and without cardiovascular diseases

MCTD manifestation	MCTD (n = 50)	MCTD/CVD^+ ^(n = 23)	MCTD/CVD^- ^(n = 27)	*P *value^a^, CVD^+ ^vs. CVD^-^
Disease duration (years)	9.56 ± 6.8 (3 to 26)	10.3 ± 6.6 (3 to 26)	8.9 ± 6.9 (3 to 25)	0.481
Polyarthritis	46 (92)	22 (95.6)	24 (88.8)	0.6123
Raynaud's phenomenon	39 (78)	22 (95.6)	17 (62.9)	0.006
Myositis	30 (60)	13 (56.5)	17 (62.9)	0.8621
Interstitial lung disease	40 (80)	19 (82.6)	21 (77.7)	0.7356
Photosensitivity	12 (24)	7 (30.4)	5 (18.5)	0.5077
Esophageal dysmotility	35 (70)	15 (65.2)	20 (74.0)	0.5480
Pulmonary arterial hypertension	12 (24)	9 (39.1)	3 (11.1)	0.0141
Lymphadenomegaly	13 (0.26)	5 (21.7)	8 (29.6)	0.7474
Serositis	19 (38)	11 (47.8)	8 (29.6)	0.2465
Secondary antiphospholipid syndrome	15 (30)	11 (47.8)	4 (14.8)	0.015
Previous venous thrombosis	13 (26.0)	9 (39.1)	4 (14.8)	0.618
Previous arterial occlusion	2 (4.0)	2 (8.69)	0	
Anti-U_1_RNP (normal: <10 U/ml)	50 (100)	23 (100)	27 (100)	
Anti-CL IgG/IgM (normal: <10 U/ml)	19 (38)	13 (56)	6 (22)	0.0195
AECA (normal: <5 U/ml)	22 (44)	15 (65)	7 (26)	0.001
SLAM (median)	5	5	4	
SLAM (mean ± SD)	6.28 ± 4.04	6.95 ± 4.08	5.44 ± 4.0	0.199
SLAM (*n*)	18 (36)	11 (47.8)	7 (25.9)	0.1438
Medication				
Nonsteroidal anti-inflammatory drugs	22 (44)	10 (43.4)	12 (44.4)	1.0
Last 2 months average dose of corticosteroids (mg/day)	3.36 ± 4.8	4.1 ± 3.4	3.1 ± 5.2	0.5306
Cumulative lifetime dose (prednisone equivalent)	14.21 (4 to 58.965)	13.0 (4 to 59.432)	15.2 (4 to 57.437)	0.674
Corticosteroids alone or combination (*n*)	28 (56)	11 (47.8)	17 (62.9)	0.3926
Patients currently corticosteroids alone *n *(%)	7 (14)	2 (8.6)	5 (18.5)	0.4295
Patients currently corticosteroids plus salazopyrine	10 (20)	5 (21.7)	5 (18.5)	1.0
Patients currently corticosteroids plus methotrexate	11 (22)	5 (21.7)	6 (22.2)	1.00
Cyclophosphamide treatment ever	37 (74)	21 (91.3)	16 (59.2)	0.0119
Antimalaric drugs treatment ever	39 (78)	19 (82.6)	20 (74.0)	0.515
Cyclosporin-A treatment ever	10 (20)	7 (30.4)	3 (11.1)	0.4804

Data presented as mean ± standard deviation, *n *(%) or median (25th to 75th percentile). AECA, anti-endothelial cell antibody; anti-CL, anti-cardiolipin; anti-U_1_RNP, anti-U_1 _ribonucleoprotein; CVD, cardiovascular disease; MCTD, mixed connective tissue disease. The systemic lupus erythematosus disease activity measure (SLAM) is a measure of disease activity [24,25]. ^a^Fischer's exact test and Student *t *test.

Concerning autoantibody profiles, all of the patients were positive for antinuclear antibodies and anti-U_1_RNP antibodies, 19 patients (38%) had anti-CL antibodies (IgG or IgM anti-CL antibodies), and 22 patients (44%) were positive for AECAs. The frequency of anti-CL-positive and AECA-positive patients was significantly higher in the MCTD/CVD^+ ^group (anti-CL, *P *= 0.0195; AECA, *P *< 0.001).

The ratio of patients taking CS at the time of the study, the cumulative median dose of CS, and other medications are also presented in Table 2.

Raynaud's phenomenon was detected in 39 out of 50 patients with MCTD. In the MCTD/CVD^+ ^group, 22 patients had Raynaud's phenomenon. Among these, 10 patients' sera contained anti-CL IgG/IgM antibodies, while 13 patients' sera were AECA-positive. An association was found between Raynaud's phenomenon and anti-CL IgG/IgM (*P *< 0.03), and between Raynaud's phenomenon and the presence of AECA (*P *< 0.02).

### Percentage FMD, percentage NMD, IMT, autoantibodies and endothelial cell markers

Anti-U_1_RNP autoantibodies, anti-CL IgG/IgM type autoantibodies, AECA and both endothelial cell markers (TM and vWFAg) were significant increased in MCTD patients versus controls and in the MCTD/CVD^+ ^versus MCTD/CVD^- ^patient groups (Table 3).

**Table 3:** Autoantibodies, endothelial cell parameters, FMD, NMD and IMT in MCTD patients with/without cardiovascular diseases

Feature	MCTD (n = 50)	Controls (n = 38)	*P *value, MCTD vs. controls^a^	MCTD/CVD^+ ^(n = 23)	MCTD/CVD^- ^(n = 27)	*P *value, MCTD/CVD^+ ^vs. MCTD/CVD^-b^
Anti-U_1_RNP (U/ml)	20.18 ± 14.6	8.02 ± 3.5	<0.001	30.3 ± 14.6	11.51 ± 7.28	<0.001
Anti-CL IgG/IgM (U/ml)	13.98 ± 12.2	6.06 ± 2.93	<0.001	21.02 ± 15.04	7.98 ± 2.95	<0.001
AECA (IU/ml)	50.1 ± 34.5	17.1 ± 8.48	<0.001	62.9 ± 26.3	39.1 ± 37.2	<0.001
Thrombomodulin (ng/ml)	12.2 ± 8.1	3.2 ± 1.3	<0.001	15.9 ± 5.2	9.0 ± 8.8	<0.001
vWFAg (%)	224.1 ± 115	89.4 ± 27.1	<0.001	311.5 ± 72.0	149.7 ± 90.0	<0.001
FMD (%)	4.76 ± 4.2	8.74 ± 5.05	<0.001	3.54 ± 2.9	5.81 ± 4.87	0.0002
NMD (%)	14.35 ± 6.67	17.16 ± 6.7	0.073	13.54 ± 6.06	15.03 ± 7.15	0.1283
IMT (mm)	0.64 ± 0.13	0.53 ± 0.14	<0.001	0.72 ± 0.11	0.57 ± 0.1	<0.001

AECA, anti-endothelial cell antibody; anti-CL, anti-cardiolipin; anti-U_1_RNP, anti-U_1 _ribonucleoprotein; CVD, cardiovascular disease; FMD, flow-mediated dilation; IMT, intima-media thickness; MCTD, mixed connective tissue disease; NMD, nitrate-mediated dilation; vWFAg, von Willebrand factor antigen. ^a^MCTD and controls, Mann-Whitney U test. ^b^MCTD/CVD^+ ^and MCTD/CVD^-^, analysis of variance test.

Percentage FMD was significant lower in MCTD patients compared with controls and in MCTD/CVD^+ ^patients versus MCTD/CVD^- ^patients (%FMD: MCTD, 4.7 ± 4.2% vs. controls, 8.7 ± 5.05%, *P *< 0.001; MCTD/CVD^+ ^vs. MCTD/CVD^-^, *P *< 0.0002). Percentage NMD did not show a difference between MCTD and controls and between the MCTD/CVD^+ ^versus MCTD/CVD^- ^patient groups.

The IMT was significantly higher both in the MCTD patients and in the MCTD/CVD^+ ^vs. MCTD/CVD^- ^patients (IMT: MCTD, 0.64 ± 0.13 mm vs. controls, 0.53 ± 0.14 mm, *P *< 0.001; MCTD/CVD^+ ^vs. MCTD/CVD^-^, *P *< 0.001).

### Correlation between flow-and nitrate mediated vasodilation, IMT and other measured parameters in MCTD patients

A significant negative correlation was found between percentage FMD and the disease duration (*r *= -0.6468, *P *< 0.001), and between percentage FMD and systolic blood pressure (*r *= -0.5423, *P *< 0.001) (Table 4). There was a positive correlation between percentage FMD and ApoA_1 _levels, and percentage FMD and PON1 activity (ApoA_1_: *r *= 0.6203, *P *< 0.001; PON1 activity: *r *= 0.5957, *P *< 0.001). Significant correlation was found between percentage FMD and AECA (*r *= -0.3075, *P *= 0.029), and between percentage FMD and inflammatory parameters such as the ESR (*r *= -0.4283, *P *< 0.001) and hs-CRP (*r *= -0.4057, *P *= 0.003).

**Table 4:** FMD, NMD and IMT in MCTD patients with/without cardiovascular disease and controls

	%FMD^a^	*P *value	%NMD^a^	*P *value	IMT^a^	*P *value
Age at investigation (years)	-0.0221	0.878	-0.6700	0.001	0.5468	0.001
Disease duration (years)	-0.6468	0.001	-0.1314	0.3628	0.1968	0.1706
Systolic blood pressure (mmHg)	-0.5423	0.001	-0.1094	0.4492	0.2220	0.1211
Diastolic blood pressure (mmHg)	0.0946	0.5133	0.1005	0.4872	-0.1597	0.2676
Serum triglycerides (mmol/l)	-0.3396	0.0158	0.2165	0.1309	-0.0411	0.7765
Serum cholesterol	-0.0459	0.7514	0.0508	0.7255	-0.0209	0.8851
Apolipoprotein A_1 _(g/l)	0.6203	0.001	-0.0805	0.5782	-0.0248	0.8640
Apolipoprotein B (g/l)	-0.3102	0.061	-0.0891	0.5997	0.0767	0.6517
Paraoxonase-1 activity	0.5957	0.001	0.1317	0.3619	-0.2789	0.0497
hs-CRP	-0.4057	0.003	-0.3207	0.023	0.7164	0.001
ESR	-0.4283	0.001	-0.3981	0.001	0.5467	0.001
Anti-U_1_RNP (U/ml)	-0.0486	0.7371	-0.0694	0.6316	0.4182	0.0025
Anti-CL IgG (U/ml)	-0.0717	0.6206	-0.0411	0.776	0.2236	0.1184
AECA (IU/ml)	-0.3075	0.029	-0.2686	0.0592	0.2890	0.0417
Thrombomodulin (ng/ml)	-0.0642	0.6577	-0.0165	0.9089	0.4823	0.00038
vWFAg (%)	-0.0122	0.9329	-0.2692	0.058	0.5443	0.001

AECA, anti-endothelial cell antibody; anti-CL, anti-cardiolipin; anti-U_1_RNP, anti-U_1 _ribonucleoprotein; ESR, erythrocyte sedimentation rate; FMD, flow-mediated dilation; hs-CRP, high-sensitivity C-reactive protein; IMT, intima-media thickness; MCTD, mixed connective tissue disease; NMD, nitrate-mediated dilation; vWFAg, von Willebrand factor antigen. ^a^Spearman rank correlation.

**Figure 1:**
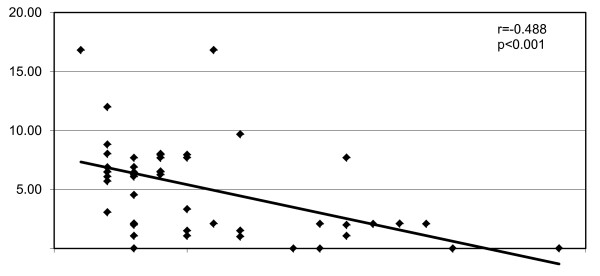
**Activity score and flow-mediated dilation in patients with mixed connective tissue disease**. Y axis: flow-mediated dilation (%). X axis: systemic lupus activity measure score (number).

We found a negative correlation between SLAM score and percentage FMD (*r *= -0.488, *P *< 0.001) (Figure 1).

NMD showed a negative association with hs-CRP (*r *= -0.3207, *P *= 0.023) and the ESR (*r *= -0.3981, *P *< 0.001). No other correlations were detected.

The IMT was significantly higher in MCTD patients compared with controls (0.64 mm vs. 0.53 mm; *P *< 0.001). The IMT was age dependent (*r *= 0.5468, *P *< 0.001), and showed an association with serum levels of anti-U_1_RNP autoantibodies (*P *= 0.0025) and AECA (*P *= 0.0417), the endothelial cell markers TM (*P *<0.001) and vWFAg (*P *< 0.001), and the ESR (*P *< 0.001) and hs-CRP (*P *< 0.001).

In our patients we found a correlation between the ESR and hs-CRP (*r *= 0.511, *P *< 0.001). In the MCTD/CVD^+ ^group, 22 patients had Raynaud's phenomenon. Evaluation of the relationship between MCTD patients with Raynaud's phenomenon and FMD is presented in Table 5. There was a significant association in MCTD patients with and without Raynaud's phenomenon and FMD. There was a negative association between FMD and MCTD patients with Raynaud's phenomenon in the CVD^+ ^and CVD^- ^groups.

**Table 5:** Correlation between flow-mediated dilation and Raynaud's phenomenon

	*n*	Correlation^a^	*P *value
MCTD with Raynaud's phenomenon	39/50	0.768	0.001
MCTD without Raynaud's phenomenon	11/50	0.25	0.235
Raynaud's phenomenon in MCTD/CVD^+ ^and MCTD/CVD^- ^patients	22/17	0.522	0.001

CVD, cardiovascular disease; MCTD, mixed connective tissue disease. ^a^Spearman correlation coefficient.

## Discussion

Cardiovascular diseases have recently been shown to be the leading causes of morbidity and mortality in patients with systemic autoimmune diseases. Some evidence shows that in autoimmune disorders, such as SLE, rheumatoid arthritis and antiphospholipid syndrome, the systemic inflammatory state itself predisposes to atherosclerotic diseases [32-35].

MCTD is a systemic autoimmune disease, involving many organs, while the most frequent, serious outcome is the development of proliferative vascular lesions in the lungs and other organs. Anti-U_1_RNP autoantibodies may have an effect on endothelial cells; in some studies on MCTD, however, the presence of antiphospholipid or anti-endothelial antibodies, in addition to anti-RNP antibodies, showed functional properties such as endothelial cell activation - changing the phenotype of endothelial cells, which become proinflammatory/procoagulant [36]. No previous studies, however, have been carried out to assess the atherosclerotic risk factors in MCTD patients.

In our study population, we investigated the traditional risk factors, in association with clinical symptoms and autoantibodies, in MCTD patients with and without cardiovascular events. This is the first study where endothelial stiffness markers are measured, including FMD, NMD and carotid IMT.

In our patients with MCTD, serum triglycerides, HDL-C and LDL-C did not differ from healthy subjects, while total cholesterol and the ApoA_1 _levels and serum PON1 activity within the liporpotein fraction were lower compared with controls.

Decreased PON1 activity and mild elevation in hs-CRP have drawn considerable interest, in relation to the development of atherosclerosis that exemplifies a low-grade chronic inflammatory process [37,38].

Decreased percentage FMD and increased IMT was found in patients with MCTD. Our results indicated that reduced FMD can clearly distinguish MCTD patients from controls and, moreover, MCTD patients with and without cardiovascular events. Decreased percentage FMD showed a close correlation with the disease duration, systolic blood pressure, and inflamatory parameters (hs-CRP and ESR).

High serum levels of hs-CRP have been shown to have a close association with CVDs, representing a link between chronic inflammation and hs-CRP with athrerosclerosis [39]. A strong correlation was described between hs-CRP and CVD events, when hs-CRP exceeded 3 mg/l [40]. In our series, both hs-CRP and the ESR were elevated in MCTD patients, escpecially in the MCTD/CVD^+ ^group, and percentage FMD showed a close negative correlation with elevated ESR. These results may suggest that MCTD patients have ongoing low-grade inflammation, and these patients have an increased risk of severe CV events. A close association between SLAM score and percentage FMD shows that the disease activity involves endothelial cell inflammation, causing endothelial cell dysfunction.

Serum concentration of anti-U_1_RNP autoantibodies and levels of AECA were elevated in the patients'sera, and both antibodies were higher in the MCTD/CVD^+ ^patients compared with the MCTD/CVD^- ^group. Furthermore, we showed that the markers of endothelial cell dysfunction, vWFAg and soluble TM were higher in patients with MCTD than in the healthy individuals.

In MCTD, high serum levels of vWFAg imply an activated state of endothelial cells and can play a pathogenic role in the development of atherothrombotic events [41]. Accordingly, in MCTD/CVD^+ ^patients the soluble TM levels were significant higher than in the MCTD/CVD^- ^group. TM is an endothelial cell activation marker, and its shedding from the endothelial cell increases the risk of cardiovascular and thrombotic events [12,42].

In patients with atherosclerotic diseases, soluble TM was elevated and its level showed correlation with the severity of coronary artery disease, and also an association with worse outcome in survivals after acute myocardial infarction [42]. Moreover, soluble TM is a good marker of disease activity in SLE with lupus nephritis [43].

vWFAg is a circulating glycoprotein, synthesized by endothelial cells - and an increased serum concentration of vWFAg has been shown to be a marker of endothelial dysfunction in scleroderma, SLE and MCTD patients with PAH [10,44].

In our patients with MCTD, percentage FMD strongly correlated with disease duration, autoantibodies to anti-U_1_RNP, AECA and anti-CL, and endothelial cell markers, such as TM and vWFAg. These data indicate that the reduced FMD is a good marker for monitoring cardiovascular complications in MCTD.

One of the earliest stages of atherosclerosis is the endothelial cell dysfunction [45]. Endothelium-dependent FMD was described previously to be significantly impaired in SLE [34], in rheumatoid arthritis [35], and in antiphospholipid syndrome [29]. In our earlier study we also found decreased FMD in patients with undifferentiated connective tissue disease (UCTD), which is an early stage of well-established connective tissue diseases [46]. Mosca and colleagues also investigated the vascular reactivity in UCTD, and found that FMD and the response to glyceryl trinitrate-mediated vasodilation were similar in UCTD patients and healthy subjects. UCTD patients were characterized, however, by having reduced response to both acethylcholine and sodium nitroprusside in the forearm microcirculation, indirectly indicating that reduced endothelium-dependent vasodilation, a peripheral microvascular risk factor, signifies UCTD [47].

In the present study we found that FMD decreased and the IMT was elevated in the MCTD/CVD^+ ^patient group, but there was no significant difference in NMD between the MCTD/CVD^+ ^and MCTD/CVD^- ^patient groups. We further described the presence of anti-CL antibodies and AECA besides anti-U_1_RNP antibodies in MCTD; moreover, serum levels of anti-U_1_RNP antibodies, anti-CL IgG and AECA were higher in the MCTD/CVD^+ ^group compared with MCTD/CVD^- ^patients.

We assume that AECA in MCTD patients could contribute to endothelial cell dysfunction. AECAs are often associated with phospholipid reactivity, present in SLE and in primary antiphospholipid syndrome.

In contrast to the data by Lima and colleagues, we found that FMD showed a correlation with the activity score [48]. Among the clinical symptoms, Raynaud's phenomenon was more frequent in the MCTD/CVD^+ ^group - our data being similar to the findings of Aizer and colleagues [49]. Interestingly, anti-CL antibodies and AECAs were more frequent in patients with Raynaud's phenomenon, and these antibodies could provoke endothelial cell damage. We assume that decreased FMD may occur as a result of continuous endothelial cell activation and impairment.

The daily doses of CS and other immunosuppressive treatment were similar in the CVD^+ ^and CVD^- ^groups. Based on our data we believe that the vascular protection is essential in patients with MCTD, especially in Raynaud's penomenon and PAH, and also aspirin and statin therapy is important from the early stage of MCTD.

Carotid IMT was significant higher in MCTD patients and showed an association with age, with disease duration and with traditional risk factors such as total cholesterol levels, systolic blood pressure and diastolic blood pressure. Ultrasound measurements of the carotid artery IMT identify early structural vascular abnormalities. An inverse correlation has been described previously between the IMT and brachial arterial FMD. Endothelial dysfunction showed a correlation with the IMT, suggesting that the carotid IMT alone was a valid surrogate marker for early atherosclerosis and had an important predicting feature in cardiovascular events in the general population [50-52].

## Conclusions

We state that, besides traditional atherosclerotic factors, autoantibodies play a crucial role in early atherosclerosis in MCTD. We believe that anti-U_1_RNP antibodies and AECA, as well as the upregulation of proinflammatory cytokines associated with vascular endothelial cell damage, may play a pivotal role in the early atherosclerotic events in MCTD.

We assume that vWFAg and TM are the most important markers of endothelial cell activation, and they impair endothelial cell functions. Finally, we found a clear decrease of percentage FMD in MCTD patients with CVD, cerebrovascular events, and peripheral arterial disease.

Our findings support the idea that the immune-mediated endothelial dysfunction, accelerated atherosclerosis present in patients with MCTD. We assume that the utilization of these serum markers and non-invasive cardiovascular measurements in the diagnosis of early atherosclerosis in MCTD provides a sufficient background for the early introduction of endothelial protection (for example, aspirin, statin) in the future management of the disease, before the development of serious cardiovascular symptoms.

## Abbreviations

AECA: anti-endothelial cell antibody; anti-CL: anti-cardiolipin; anti-U_1 _RNP: anti-U_1 _ribonucleoprotein; ApoA_1_: apolipoprotein A; BMI: body mass index; CVD: cardiovascular disease; CS: corticosteroids; dsDNA: double-single DNA; ELISA: enzyme-linked immunosorbent assay; ESR: erythrocyte sedimentation rate; FMD: flow-mediated dilation; HDL-C: high-density lipoprotein-cholesterol; hs-CRP: high-sensitivity C-reactive protein; IMT: intima-media thickness; LDL-C: low-density lipoprotein-cholesterol; MCTD: mixed connective tissue disease; NMD: nitrate-mediated dilation; PAH: pulmonary arterial hypertension; PON1: paraoxonase-1; SLAM: systemic lupus activity measure; SLE: systemic lupus erythematosus; TM: thrombomodulin; UCTD: undifferentiated connective tissue disease; vWFAg: von Willebrand factor antigen.

## Competing interests

The authors declare that they have no competing interests.

## Authors' contributions

PSo performed acquisition and analysis of the data. DB and PSz performed interpretation of the data and manuscript preparation. MTM, HD, IC and AH performed interpretation of the data and drafted the manuscript. GP and GS performed analysis and interpretation of the data. EB gave final approval of the version to be published. All authors read and approved the final manuscript.
